# Validating overcrowding measures using the UK Household Longitudinal Study

**DOI:** 10.1016/j.ssmph.2019.100439

**Published:** 2019-06-25

**Authors:** Noriko Cable, Amanda Sacker

**Affiliations:** Department of Epidemiology and Public Health, University College London, 1-19 Torrington Place, London, WC1E 6BT, United Kingdom

**Keywords:** Overcrowding, Person per room ratio, Bedroom standard, Modified bedroom standard, Validity, UK household longitudinal study

## Abstract

Overcrowding has been regarded as indicating material deprivation and treated as a proxy measure for individual socioeconomic status. Conventionally, ‘persons per room’ (PPR) has been employed to identify overcrowded households in UK survey data, though the ‘bedroom standard’ (BS) approach or the ‘modified bedroom standard’ (MBS) approach has been thought to capture overcrowded households better. Little is known about which overcrowding measure will perform well in regard to construct and face validity. In this work, associations between three overcrowding measures and socioeconomic (income and household tenure status) and health (satisfied with health and GHQ12) indicators were assessed, using the UK Household Longitudinal Study Wave 6 data.

PPR, BS and MBS were derived using relevant housing grid information and housing information from the dataset, which were aggregated at a household level (N = 18,848). Raw scores were categorised into ‘under occupied (rooms < people)’, ‘balanced (rooms = people)’, ‘overcrowded (rooms < people)’ according to an established cut-off point for each overcrowding measure. Kappa coefficient was used to assess the level of agreement between overcrowding measures. Construct validity of the measures were tested against log-transformed household equivalised income and housing tenure status as well as with each component of overcrowding measures. Using individual data (N = 38,455), face validity of the overcrowding measures was tested against satisfaction with health and mental health indicated by GHQ12.

Each overcrowding measure has a fair agreement with the others (kappa = 0.44, p<0.001). All overcrowding measures were significantly correlated with income and household tenure in a similar manner. However, components of overcrowding measures were associated differently to these socioeconomic indicators, while they were better correlated with satisfaction with health compared to GHQ12, showing a complex aspect of overcrowding measures.

In sum, use of PPR as a socioeconomic indicator is reasonable. However, given the complexity of the mechanism of health inequalities, the relevant household information is required to understand the link.

## Highlights

• Two newer overcrowding measures moderately agreed with the person per room ratio (82).
• All three overcrowding measures were similarly related to other SEP indicators (81).
• Components of the overcrowding measures uniquely correlated with the SEP indicators (86).
• Overcrowding's complexity suggests relevant household data is needed in surveys. (81).

## 1 Background

Overcrowded housing has been equated with social disadvantages for a long time, given the strong associations with non-tenure housing status (Ellaway & Macintyre, 1998; Macintyre et al., 2003) and financial adversity (Bartley, Kelly, & Sacker, 2012). Overcrowding has been linked with the material pathway to health inequalities (Bartley, 2017) because of overcrowding being viewed as household related material resources such as housing tenure and income (Galobardes, Shaw, Lawlor, Lynch, & Davey Smith, 2006).

Material based measures are components of socioeconomic position (Krieger, Williams, & Moss, 1997) that distinguish socially advantaged groups from disadvantaged (Galobardes, Lynch, & Smith, 2007). Given a direct link between housing and material deprivation or hardship (Krieger et al., 1997), use of overcrowding as a material aspect of socioeconomic position is justifiable.

Whilst sharing an aspect of socioeconomic position, overcrowded housing plays the role of a structural determinant for children's (Kelly et al., 2013) and adults' (Cable, Kelly, Bartley, Sato, & Sacker, 2014) respiratory health because of the damp and mould commonly found in such housing.

Overcrowding has been assessed using a simple ratio between numbers of people in the household and rooms – namely, a person per room ratio (PPR) (Reynolds, 2004), with a PPR of over 1.0 being regarded as overcrowded. Criticising this conventional measure of the overcrowded household, the bedroom standard (BS) approach that applies a set of rules to allocate a bedroom to each household member (Communities and Local Government, 2010) was suggested to adequately capture overcrowded households, while Shelter (2006) even suggested modifying the age threshold used in the bedroom standard approach (MBS) to identify overcrowded households accurately.

To date, it has been little addressed how closely these overcrowding measures share a constructive property of socioeconomic conditions, i.e. material resources with one another. This study aims to address the validity of these overcrowding measures using the UK Household Longitudinal Study through answering these two research questions:

• *What is the level of agreement between the three overcrowding measures?*
• *Does the association between overcrowding and income, housing tenure, and physical and mental health measures vary according to the three overcrowding measures?*

## 2 Methods

### 2.1 Dataset

Available household data (N = 18,848) and individual data (N = 38,455) from adults 16 and over who participated in Wave 6 of Understanding Society (UK Longitudinal Household Study, UKHLS) were used for the study. UKHLS has annually been collecting population representative data in the UK from a selected 40,000 households since 2009(Institute for Society and Economic Research, 2014). Individual data regarding household composition (numbers of people in the household, gender and age), numbers of bedrooms and other rooms, housing tenure and household income were aggregated at the household level to generate relevant variables, described below.

### 2.2 Overcrowding measures

Three approaches (*PPS, BS, MBS*) were used to indicate overcrowding.

The *persons per room (PPR)* approach divides the number of household occupants by the number of rooms, excluding kitchen or bathrooms, to indicate overcrowding. *The bedroom standard (BS)* approach (Shelter, 2006) allocates a bedroom for: each couple, each single person aged 21 and over. A shared bedroom is allowed for each pair of same-sex adolescents (10–20 years) and each pair of the same or opposite sex children (under 10 years old). For a remaining unpaired same-sex adolescent and children, sharing a bedroom is allowed, but if their sex is different, a separate bedroom is required for each child. The total numbers of expected bedrooms is calculated. If the number is greater than the actual number of bedrooms, the home is considered overcrowded. *The modified bedroom standard (MBS)* approach (Shelter, 2006) applies the same rules but with children under 8 years old, adolescents 8–17 years old, and adults 18 years old and over.

Given the numeric relationships derived from available rooms and numbers of people in the household, all overcrowding measures were classified into (1) under occupied (rooms > people), (2) balanced (rooms = people), and (3) overcrowded (rooms < people).

#### 2.2.1 Validating variables

Household equivalised income was derived using household income and a squared root of numbers of people in the household. A natural log of the value was taken to accommodate skewed distribution. Information about housing tenure (i.e. house ownership) of each household was categorised into 0 being ‘rented’ and 1 being ‘has ownership’.

### 2.3 Analysis

Information about overcrowding, income, housing tenure were aggregated at a household level. All analyses were conducted with Stata v15.1 (Stata Corp, College Station, TX). Agreement between PPR and the two other overcrowding measures was assessed descriptively and using kappa correlations (McHugh, 2012). Next, the associations between each overcrowding measure and other related socioeconomic indicators – namely, income and housing tenure – were examined for construct validity, either applying regression for the associations with income or logistic regression for the associations with housing tenure. Additionally, the individual component of overcrowding measures that are numbers of bedrooms and other rooms and number of people in the household (adults and children for BS and MBS) were entered to assess associations with income and housing tenure.

Face validity of overcrowding measures was assessed through examining overcrowding measures’ associations with physical health indicated by 7 item self-reported satisfaction to health and mental health indicated by a total score on the GHQ 12 questionnaire, applying regression analyses. Conventionally analysing the UKHLS datasets requires to accommodate complex survey design and missing information [9] for which we applied the svy command from Stata and a cross-sectional weight, either household or individual, to relevant analyses.

## 3 Results

In available cases of households (N = 18,848) in the UK, many were solo living (31.40% 95%CI = 30.56–32.26) or 2 occupants (32.46%, 95%CI = 31.67–33.26), and it was infrequent to have more than 5 occupants (5.17%, 95%CI = 4.82–5.54%) in the same household. Tabulation of overcrowding status by PPR against overcrowding status by BS or MBS (Table 1) showed moderate agreement in under occupied (nearly 80% for both BS and MBS), balanced (68% for both), and overcrowded (55% and 59%, respectively). About 20% of the households identified as balanced by PPR were categorised as under occupied by BS or MBS, while approximately 40% of households identified as overcrowded by PPR were categorised as balanced by BS or MBS. On the other hand, around 15–18% of the households identified as balanced by PPR were categorised as overcrowded by BS or MBS.

**Table 1:** Tabulation of overcrowding status with PPR against the overcrowding status in % with 95%CI by BS or MBS in the UKHLS household at the Wave 6 (N = 18,848).

PPR	Under occupied		Balanced		Overcrowded	
	BS	MBSw	BS	MBS	BS	MBS
Under occupied (n = 15,937.68)	79.37 (78.97,80/48)	78.99 (78.22,79.74)	19.87 (19.14,20.06)	20.51 (19.77,21.27)	0.39 (0.29,0.51)	0.48 (0.38,0.62)
Balanced (n = 3362.15)	16.16 (14.26,18.25)	13.54 (11.82,15.47)	68.59 (65.89,71.19)	68.50 (65.77,71.11)	15.23 (13.06,17.70)	17.95 (15.66,20.49)
Overcrowded (n = 2,449.89)	1.47 (0.77,2.82)	0.81 (0.34,1.87)	43.54 (39.68,47.47)	39.22 (35.50,43.08)	54.97 (51.05,58.84)	59.96 (56.00,63.72)

Note: Results were weighted for households. PPR = person per room ratio, BS = Bedroom Standard, MBS = modified bedroom standard.

Overall, kappa coefficients confirmed that PPR was in moderate agreement with BS (Agreement = 77.17%, kappa = 0.44, p<0.001) or MBS (Agreement = 76.74%, kappa = 0.44, p<0.001). It is possible that large numbers of households with one or two occupants could have driven the level of agreements with BS or MBS. Sensitive analysis excluding those households showed a similar level of agreement with BS (Agreement = 69.06%, kappa = 0.47, p<0.001) and MBS (Agreement = 68.05%, kappa = 0.47, p<0.001).

Regression analyses showed all overcrowding measures were significantly negatively associated with log-transformed income, while logistic regression analyses showed a similar association between overcrowding measure and household tenure status (Table 2). Each component of overcrowding measures was associated differently with household tenure. Numbers of bedrooms were negatively associated with housing tenure status, while numbers of other rooms showed the opposite. Similarly, numbers of children were negatively associated with household tenure status, while numbers of adults in the household were positively associated with the same variable. Regression analyses using individual data showed anticipated associations between overcrowding measurement and physical or mental health outcomes. All overcrowding measures was associated with the physical health outcome in a linear manner, indicating more insufficient space was related to poorer health. In contrast, diminishing housing space did not show similar associations with GHQ12 score, especially with the MBS (see Table 3).

**Table 2:** Bivariate association between overcrowding measures and income (log-transformed)^a^ and housing tenure^b^ indicated by coefficients with 95%CI (N = 18,848).

	Income^a^ (transformed)	Housing tenure^b^
Person per room ratio (PPR)^c^		
Balanced	−0.61 (−0.68,-0.54)	−1.19 (−1.32,-1.07)
Overcrowded	−0.51 (−0.58,-0.44)	−1.62 (−1.79,-1.44)
Bedroom standard (BS)^c^		
Balanced	−0.34 (−0.38,-0.29	−1.84 (−1.93,-1.75
Overcrowded	−0.38 (−0.47,-0.30	−2.22 (−2.40,-2.02
Modified bedroom standard (MBS)^c^		
Balanced	−0.35 (−0.41,-0.31)	−1.81 (−1.90,-1.73)
Overcrowded	−0.42 (−0.50,-0.34	−2.10 (−2.28,-1.93
Number of bedrooms	−0.06 (−0.08,-0.04	1.04 (0.99,1.09
Numbers of other rooms	0.04 (0.01,0.06	1.32 (1.24,1.40
Numbers of total people living in the household	−0.31 (−0.33,-0.29	0.02 (−0.02,0.04
Numbers of adults living in the household (BS)	−0.29 (−0.33,-0.27	0.65 (0.58,0.73
Numbers of adults living in the household (MBS)	−0.30 (−0.32,-0.27	0.49 (0.42,0.55
Numbers of children living in the household (BS)	−0.39 (−0.41,-0.36	−0.22 (−0.25,-0.19
Numbers of children living in the household (MBS)	−0.39 (−0.41,-0.37	−0.23 (−0.27,-0.20

Note: Results were weighted.

a Regression coefficient.

b Logistic regression coefficient.

c Under occupied is the reference category.

**Table 3:** Bivatiate associations between overcrowding measures and self-reported satisfaction to health and the GHQ12 total score, using the UKHLS individual data. (N = 38,455).

	Satisfaction to Health	GHQ12
	Mean (95%CI) 4.32(4.25,4.39)	Mean (95%CI) 10.08(10.73,10.88)
PPR^a^		
Balanced	−0.82(-1.10,-0.55)	0.38(0.14,0.62)
Overcrowded	−1.25 (−1.70,-0.73)	0.38(0.03,0.74)
BS^a^		
Balanced	−0.75(-0.91,-0.59)	0.69(0.51, 0.87)
Overcrowded	−1.85(-2.61,-1.10)	0.72(0.33,1.10)
MBS^a^		
Balanced	−0.73(-0.88,-0.57)	0.72 (0.55,0.90)
Overcrowded	−1.62(-2.27,-0.97)	0.55 (0.20,0.90)

Note: Results were weighted.

a Underoccupied is the reference category.

## 4 Discussion

The validity and psychometric properties of three overcrowding measures (PPR, BS, MBS) were assessed using kappa coefficients and bivariate associations against other socioeconomic indicators such as household equivalised income and housing tenure status. The findings show that each measure is similar to both the others and able to serve as a material based SEP indicator.

The tabulation of overcrowding status in PPR against BS or MBS showed an under or overestimation of overcrowding with these measures. These discrepancies in distributions of overcrowding status across the measures are due to the method of derivation. Overestimation of overcrowded households by PPR compared to BS or MBS is explained by differences in room allocation; PPR allocates a room to each person in the household, while BS or MBS does not allocate a bedroom to each child in the household. On the other hand, PPR includes all rooms apart from kitchen and toilet, while BS and MBS consider bedrooms only, leading to underestimation of overcrowded households in PPR. Nevertheless, this proportion is small, and moderate kappa coefficients between these measures, with or without households with one or two occupants suggest those measurements are comparable.

Additionally, all overcrowding measures were similarly correlated with income or household tenure status, supporting the validity of those measures, while face validity was provided testing against health measures. PPR was not strongly correlated with housing tenure, compared to the associations with housing tenure found in BS or MBS. This can be explained by the opposite direction of associations between numbers of adults and children with household tenure status, diluting the degree of associations between them.

### 4.1 Study limitations and implications

In earlier days, the definition of ‘rooms’ in the UK was different in Scotland and England (Davey, Butler, & Goldstein, 1972). However, the protocol of data collection for the UKHLS is the same across all countries (Institute for Society and Economic Research, 2014); overcrowding measures were comparable in that sense. Rich data on numbers of the households and information made comparisons across overcrowding possible which is the main strength of this study. Given the respectable kappa coefficients and associations with income or housing tenure status, choosing any of the overcrowding measures is likely to yield similar results. Results from validity tests also suggest PPR is a suggested overcrowding measure in survey settings if the resources and time are limited.

We found a certain degree of disagreement in overcrowded statuses between the measures. Having information on the size of each room, i.e. person /m^2^ would have added precision in determining overcrowded households and in testing the sensitivity and specificity of each overcrowding measure. Unfortunately, such information was not collected in the UKHLS. Moreover, assessment on face validity of the overcrowding measurements were examined the cross-sectional association with physical and psychological health measures. Patterns housing composition such as single parent household can be complex, especially in concerning individual mental health. However, having insufficient space is clearly associated with dissatisfaction with health, suggesting usefulness of these three overcrowding measures in exploring social gradients in health through the material pathway.

In the survey setting, it is practical to avoid collecting excessive information unnecessarily. Nevertheless, having an adequate space is necessary to meet individual needs for privacy (Davey et al., 1972) and there was a possible link between overcrowded households and chronic stress (Riva et al., 2014). Moreover, finding complex associations between overcrowding measures, components of overcrowding, and health measures suggests that researchers need to capture relevant household information to test how an overcrowded household determines poor health over time and across the life course.

## 5 Conclusion

Use of PPR, BS or MBS can capture the characteristics of an overcrowded household in a similar manner. While use of PPR as a SEP measure appears to be reasonable, the choice of overcrowding measure depends on the mechanisms between overcrowding and health outcomes, and relevant household information is required to understand the mechanisms.

## Ethical statement

The data used are publically available via UK data archive, which does not require any ethical assessment for the academic research use. This work is supported by the Cohort and Longitudinal Studies Enhancement Resources (CLOSER) Innovation Fund 2 which supported by the UK Economic and Social Research Council. .
